# Climate change, biofuels and eco-social impacts in the Brazilian Amazon and Cerrado

**DOI:** 10.1098/rstb.2007.0030

**Published:** 2008-02-11

**Authors:** Donald Sawyer

**Affiliations:** 1 Centre for Sustainable Development (CDS), University of Brasília (UnB)Brasília DF 70070-914, Brazil; 2 Instituto Sociedade, População e Natureza (ISPN)SCLN 202, Bloco B, Sala 101, Brasília DF 70832-525, Brazil

**Keywords:** Amazon, Cerrado, frontier, biofuel, impacts

## Abstract

While global technical progress is relatively linear, there is wide variation in its environmental and social impacts at the local level, with cycles of expansion and retraction or boom and bust, of long or short duration. Analysis of previous open-ended stages of extraction and agro commodities in the Amazon indicates a general gravitational trend for technical progress to increase productivity and permit transformation of increasingly generic forms of material or energy, rather than relying on the specific physical or chemical properties provided by nature. While increased demand favours frontier expansion in the periphery when there is no other alternative, technical progress ultimately favours spatial reconcentration of production in central countries. The agroenergy stage now beginning involves rapid frontier expansion and offers various environmental and economic opportunities, but also generates a series of negative ecosystemic and socio-economic impacts, which are both direct and indirect, for tropical regions. The Amazon and the Cerrado are particularly vulnerable. Interacting with climate change and land use, the upcoming stage of cellulosic energy could result in a collapse of the new frontier into vast degraded pasture. The present and future impacts can be mitigated through crafting of appropriate policies, not limited to the Amazon, stressing intensified and more sustainable use of areas already cleared, minimizing new clearing and consolidation of alternatives for sustainable use of natural resources by local communities. Coping with these scenarios requires knowledge of complex causal relationships.

## 1. Introduction

This article examines interactions among climate change, political-economic interventions and technical progress, focusing on the impacts of biofuels in the Amazon and Cerrado regions in Brazil. The fates of the Amazon and the Cerrado are intertwined, but the importance of the Cerrado, a wooded savannah, is practically unknown, and the threats it faces are neglected (Scariot *et al*. 2005). Understanding the diverse impacts of biofuels depends on contextualized analysis in space and time, i.e. historical socio-ecosystemic analysis (Sawyer 2001). In this article, technical progress is considered to be a major driving force, with outcomes that are not easily predictable. While it is relatively linear, although neither neutral nor deterministic (Feenberg 2005), there is considerable variation in its environmental and social impacts at various locations and over time. If causal relationships are better understood and taken into account in the design of policy, some unpleasant surprises might be avoided.

Direct and indirect environmental impacts of agroenergy production in the short run will almost certainly include increased deforestation, which will interact with dieback due to climate change. Biofuels also generate various social impacts. The adoption of technology for cellulosic ethanol in the near future could undermine the agroenergy frontier. Coping with the complexity of the interactions and concomitant centrifugal and centripetal spatial trends requires ‘sustainability sciences’ for design of appropriate policies to reconcile, to the extent possible, the diverse outcomes, maximizing opportunities and minimizing risks.

The complexity and apparent unpredictability can be better understood in an historical perspective. The historical record shows cycles of boom and bust, expansion and retraction, centrifugal and centripetal trends or ‘spread’ and ‘backwash,’ with various unexpected and inconvenient consequences (Hirschman 1958; Furtado 1963; Myrdal 1971; Sawyer 1984, 1985). For present purposes, the past and the near future have been divided into four open-ended stages of technological change in the Amazon and Cerrado (extraction, agro commodities, agroenergy and cellulosic energy), which start at certain points in time, but overlap rather than ending entirely.

### (a) Stage 1: extraction (1616-)

After Portuguese attempts to plant sugar cane in the Amazon failed, due to the lack of slave labour and the attractions of the exuberant tropical forest, European colonial expansion and the Industrial Revolution favoured extraction of Amazon natural resources with very specific physical and chemical properties, known as ‘backlands drugs,’ e.g. spices, medicinal plants and cacao, as well as Brazil nuts and rubber (Santos 1980). Such extraction kept the ecosystem relatively intact, at least when extraction did not involve extermination, as it did in the cases of turtles or *caucho*. Wild collection was and continues to be highly sustainable in terms of maintaining ecosystem functions. In the first half of the twentieth century, the shift from extraction in the Amazon to production in other countries, as in the emblematic case of rubber planted in Malaya or produced synthetically from petroleum in developed countries, led to economic collapse in the Amazon and spatial reconcentration at the global level (Sawyer 1979; Bunker 1985; Hecht & Cockburn 1989).

### (b) Stage 2: agro commodities (1960-)

Subsequently, facilitated by investments in transportation infrastructure and green revolution technology, rice, cattle and soya frontiers expanded from central and northeastern Brazil to the north and west (Foweraker 1981; Sawyer 1990; Schmink & Wood 1992; Margulis 2004; Veiga *et al*. 2004). This centrifugal expansion contrasted with the pattern of spatial concentration or reconcentration usually associated with urban industrial development, due to economies of scale and agglomeration (Singer 1976; Storper 1991; Diniz & Lemos 2005).

The replacement of extraction by agribusiness and commodity production, primarily for food crops and cattle for domestic consumer markets, as well as associated land speculation, generated deforestation reaching a record of 29 000 km^2^ yr^−1^ in 1994–1995 (Fearnside 2005). After 1990, there was a boom in soya and after 2000, a boom in beef, both intended primarily for global markets (Miragaya 2007). This frontier expansion, i.e. spatial deconcentration, was made possible by technological change in production, processing and transportation.

After 1988, clearing in the Amazon, seen from outer space and dramatized by the murder of Chico Mendes, attracted the attention of the world. It prompted new international initiatives, such as the Pilot Programme to Conserve the Brazilian Rain Forest, supported by the G-7, starting in 1992, and new national policies, such as the Action Plan to Prevent and Control Deforestation in the Legal Amazon, starting in 2004. At first, the main global concern, internalized in Brazil, was with loss of biodiversity, which led to the establishment of the National Biodiversity Policy in 2002 (Decree 4339/2002). As of 2007, as seen in this volume, carbon emissions from deforestation in the Amazon have become the main cause of global concern. This maintains the recent emphasis on saving natural resources, rather than exploiting them directly, in contrast to the previous pattern of deconcentration. Thus, although it was not stronger than market forces, policy started to favour reconcentration of production in developed regions.

### (c) Stage 3: agroenergy (2007-)

Now, faced with global warming caused primarily by consumption of fossil fuels in developed countries as well as political insecurity in many countries that produce petroleum, the North is seeking alternative supplies of energy. Biofuel, i.e. ethanol or bio-diesel, is considered the best alternative (Farrell *et al*. 2006). Biofuel grants Brazil and other tropical countries in the global South a new role as producers of agroenergy, because land and labour have low costs and natural conditions (sunlight, temperature and rainfall) are favourable for photosynthesis year round. Government and business are also keen to invest (MME 2004; MAPA 2005; Jank 2007; Rothkopf 2007; UNICA 2007).

Agroenergy reflects the fact that, in addition to raising yields, continued technical progress has now made it possible to use increasingly generic forms of material or energy, rather than relying on the specific physical or chemical properties provided by nature, through extraction, or even through agricultural production (cf. Sawyer 1985; Goodman *et al*. 1987; Piasentin & Ruivenkamp 2006). So far, ethanol is produced from carbohydrates (starch in maize and sugar in cane), while bio-diesel is made from various plant oils, primarily soya and palm. Efficient large-scale production of carbohydrates and oils generally involves monocultures. During the stage now beginning in Brazil, this leads to further deforestation and conversion from pasture to crops, i.e. rapid frontier expansion in the Centre-West and North regions and revitalization of the pattern of deconcentration.

### (d) Stage 4: cellulosic energy (2015-)

By the middle of the next decade, according to various predictions, it will be economically feasible to produce biofuel from cellulose, i.e. generic biomass, rather than carbohydrates or plant oils (Hill *et al*. 2006; Bourne 2007; Wald 2007). Given the historical record reviewed previously, on the one hand, and the availability of wood chips, grasses, agricultural residues and other low-value, generic biomass in developed regions and countries, on the other, it can be inferred that spatial reconcentration of biofuel production is likely to occur on land closer to markets, including marginal land not suited for grain or cane. This gravitational movement is possible because low-value biomass is abundant in developed countries and regions, and more can be produced on marginal land. Industrial technology is also more developed. The key issue is how much energy is required to transform cellulose. If energy costs are not too high, and unless production and transportation costs can be reduced drastically, production of biofuel in peripheral regions or countries could lose its competitive advantage and become unprofitable (cf. Sawyer 1985). High-tech agricultural production of specific raw material like soya and sugar cane may be replaced by high-tech industrial processing of generic plant biomass, using the whole plant, not just its sap or seed. This is more likely to occur in developed countries or regions. Of course, the use of other sources of energy for automobiles, like hydrogen or electricity, would also have similar negative impacts on biofuel production in the tropics.

## 2. Opportunities and risks

The biofuel solution offers various global opportunities, heralded by various businesses and governments, especially in recent months (sources above), such as the potential to generate environmental benefits by reducing emissions of CO_2_ from automobiles or at least slowing their growth (Wald 2007). For developed countries, biofuel is also seen as part of a strategy of energy independence, i.e. decreased dependence on oil from the Middle East, Russia or Venezuela (Carelli 2007). It is also a convenient justification for providing subsidies to the farm sector, a powerful interest group (Crooks & Harvey 2007). For developing countries, especially Brazil, there are promises of generation of employment and income, opportunities for foreign investment, regional development in depressed areas, new tax and foreign exchange revenues, sale of technology and technical cooperation with Africa, as has been widely reported recently in the press and official documents (see UN 2007).

However, there are also various questions about costs and risks, especially for the global South. To begin with, the production and distribution of biofuels, when the entire life cycle is considered, still require considerable use of fossil fuels for fertilizer production, transportation of inputs and labour, manufacture and operation of farm machinery, processing of raw material and transportation to markets, among other energy needs (UN 2007). Thus, they may offer few if any net benefits in terms of emissions of CO_2_, unless there are significant gains in productivity in the fields and in efficiency of processing and distribution. Inevitably, there are also emissions of CO_2_ from clearing of land not already farmed, as well as emissions of N_2_O, a potent greenhouse gas, from nitrogen in fertilizers (Hill *et al*. 2006).

### (a) Biofuels, the Amazon and Cerrado

To the extent that the biofuel response to climate change is limited to production and use of bio-diesel from soya beans or ethanol from sugar cane or maize, without due caution, it may have strong negative impacts on the Amazon and other tropical biomes, especially the Cerrado, interacting with climate change. As seen in other pieces in this volume, climate change may cause vast dieback of the Amazon forest or reduction to scrub.^1^ On the other hand, little or no attention has been given to even greater past and current clearing in the Atlantic Forest and the Cerrado, global biodiversity hotspots that, according to various climate models, would receive less water vapour from the Amazon (Machado 2004; Marengo 2006). River flow from the Cerrado to neighbouring biomes could also be affected (Lima & Silva 2002).

There is now two or three times as much annual deforestation in the Cerrado as the Amazon, i.e. 22 000–30 000 km^2^ yr^−1^ in the Cerrado, as compared with 13 100 km^2^ in 2005–2006 and 9600 in the Amazon in 2006–2007 (Machado 2004; R. Machado 2007, personal communication; ISPN 2007). The accumulated deforestation in the Cerrado is between 800 000 km^2^ and 1 600 000 km^2^, depending on the estimate, as compared with 700 000 km^2^ in the Amazon, which is nearly twice as large (Buschbacher 2000; Machado 2004; Sano 2006). The woodland and savannah matrix of the Cerrado is especially vulnerable because it has less protection and is considered to have low value, even offering an alternative to deforestation in the Amazon (Bispo & Privado 2005; ISPN 2005, 2007; Rede Cerrado 2006; Ferraz 2007). The Brazilian government intends to exclude sugar cane from the Amazon and the Pantanal wetlands, as if there were no problems in the Cerrado (Paraguassu 2007).

### (b) Ecosystemic effects

The direct and indirect negative impacts of biofuels can be ecosystemic, causing impacts on biodiversity, water and carbon, or social, including economic and political dimensions, in various ecosystems. Scientific studies are needed to verify the reports and allegations about negative impacts that mushroomed in 2007,^2^ as summarized below, and to quantify impacts of various crops and production technologies in different locations.

Depending on the crop, location, previous land use and technology, the direct ecosystemic effects of expansion of soya and cane monoculture may include, according to various sources cited by Rodrigues & Ortiz (2006) and Honty & Gudynas (2007), among others, damage to biodiversity, soils, water resources and the atmosphere. Obviously, destruction of biodiversity occurs when forest or savannah land or land undergoing regeneration is cleared. Not so obviously, biodiversity is also reduced when mixed farming systems are replaced by monoculture landscapes. Owing to the effects of wind and water, soil erosion occurs when natural vegetation is removed, unless minimum tillage or integrated crop–livestock systems are used. Soil fertility is also reduced due to contamination, compaction and loss of organic matter.

Cane production and processing consume huge quantities of water, as much as 4:l per litre of ethanol (Gabeira 2007). Clear fields accelerate run-off, reducing infiltration of water into the soil and aquifers, which may also affect water supplies in downstream reservoirs during the dry season (Lima & Silva 2002). Water is polluted with pesticides and nitrogen and phosphorus from fertilizers (Hill *et al*. 2006). Clearing woodland, including the eventual decomposition of underground carbon in roots, generates massive emissions of CO_2_ into the atmosphere. There are also greenhouse gas emissions of N_2_O from fertilizer use. Smoke and ashes from the widespread practice of burning sugar cane fields before manual cutting cause local atmospheric pollution. There is also pollution due to pesticides sprayed from the air (sources cited above).

### (c) Sociosystemic effects

Various negative social, economic and political impacts of biofuel production within the socio-economic context of Brazil have also been identified in these sources, the press and electronic media. First of all, concentration of land tenure continues or is exacerbated, since monocultures of cane and soya beans require large areas for mechanization and, especially in the case of sugar-cane processing, for sufficient scale. There is also concentration of income, given that producers and processors make large profits, while workers are displaced or earn low wages (Barrocal 2007; Oliveira 2007).

While soya beans eliminate employment, sugar cane involves temporary semi-proletarianization. Although mechanization is underway, 80% of sugar canes harvested in Brazil are cut manually by approximately 1 million seasonal workers (Lima 2007). Work conditions are unhealthy, shortening working life and even causing death from exhaustion, due to manual cutting of sugar cane involving tens of thousands cutting strokes per day (Barrocal 2007; Lima 2007; Silva 2007). Displacement and seasonal labour involve physical and cultural destruction of multifunctional family farms and traditional communities (Bispo & Privado 2005; Rede Cerrado 2006; ISPN 2005, 2006, 2007).

Finally, although cane and soya in Brazil are different from maize in the USA, food prices are rising owing to competition for land and capital in the current context of expanding markets for grain and beef (CONSEA 2007; Economist 2007; FAO 2007). This benefits farmers, and could even help them adopt more sustainable practices, but it stimulates frontier expansion and does not benefit the population at large.

### (d) Inter-regional connections

Analysis of these impacts should take inter-regional and even international interactions into account. In addition to the direct and indirect effects of expansion of soya and cane monoculture, extensive cattle-raising is being displaced to frontier areas, where its area is multiplied, generating strong pressures for large-scale deforestation. Ranchers who sell their land to planters of soya or cane can purchase areas 10 times as large on the frontier, owing to the strong differential in land prices. The average price of land in the North region is seven times less than in the South and the differential is increasing (Hackbart 2007), while differentials between southern farmland and uncleared land on the frontier are greater than those between the regional averages.

Although there are restrictions regarding clearing and planting soya and cane in the Amazon, there are no specific national or international policies or actions to limit the expansion of cattle-raising. The soya moratorium negotiated by Greenpeace in 2006 was restricted to traders agreeing not to purchase soya from newly cleared areas in the Amazon in the next 2 years (ABIOVE 2007; Kaufman 2007). There is no monitoring of deforestation outside the Amazon.

Deforestation will interact with possible dieback of rainforest and desertification in sub-humid areas due to climate change. As seen in this volume, deforestation accelerates dieback owing to fragmentation, edge effects, increased flammability and multiplication of sources of ignition, while dieback provides feedback for higher temperature and lower humidity. Thus, some government policies, international investments and even some NGO positions that ignore the Cerrado tend to favour the worrisome scenario of anthropogenic climate change interacting with deforestation—itself driven in part by the biofuel response to climate change—in both the Cerrado and the Amazon.

There may also be risk that increased economic dependence will lead to increased political influence on the part of consumer countries that become dependent on the new sources of energy. Sugar cane plantations involve large long-term investments that require security and political stability, but they are also notorious for being socially unstable or disruptive, as in Cuba and the Northeast of Brazil (Oliveira 2007).

Owing to technical progress permitting production of cellulosic ethanol, the worst impacts of biofuels may come in the next decade, due to regional reconcentration mentioned with regard to Stage 4. The apparent biodiesel and alcohol boom in Brazil could collapse into an empty frontier, not unlike the collapse of the rubber economy, except for the dimensions of its devastation. The result could be degraded land subject to fire, abandoned infrastructure, bankrupt farmers and unemployed seasonal workers. The Cerrado and the Amazon, no longer needed for production of carbohydrates or plant oils, could become vast degraded pastures, as might be predicted on the basis of the model of land use of von Thünen (Hall 1966). Widespread low-intensity backlands ranching would also involve increased production of methane, another potent greenhouse gas. Induced both directly and indirectly by climate change, dieback could result in economic bust, social unrest and political instability. With less rainfall being transmitted inland, desertification might result. Thus, by a different route, more global than the one they envisaged, the scenario suggested by Goodland & Irwin (1975)—from Green Hell to Red Desert—could come true, at least in part, but not limited to environmental change.

## 3. Policy implications

If policy makers are aware of probable global trends, their local impacts can be mitigated, without entirely missing opportunities. Crafting policy for more sustainability and justice in the short and long run requires that the various costs, benefits and externalities be considered, in different places and over time, taking into account the nonlinear but non-random consequences and differences between central and peripheral locations.

First of all, many environmentalists and governments argue that responses to climate change should not be limited to simply replacing fossil fuel with biofuel, but should start with changes in consumption patterns in developed countries, even if only beginning the process, which involves high costs. Extending this logic, ‘carbon neutrality’ through compensation for avoided emissions (e.g. planting trees) should be part of adjustment, rather than being a substitute for reduced consumption.

Since change in transportation logistics will take time, given that established production and trade networks and sprawling settlement patterns are rigid and difficult to change any time soon, expansion of agroenergy production in Brazil is inevitable in the short run. Biofuels should be produced in sustainable ways in areas that have already been cleared and have low productivity, mostly old pastures, minimizing new clearing. This requires the development of technology to lower the costs of recovery of degraded land and mitigate the impacts of erosion and pollution. It also requires policy measures to intensify land use, improve environmental and social law enforcement and increase the costs of deforestation.

Another approach to mitigation of possibly disastrous impacts of biofuels would be strengthening alternatives for family and community livelihoods in sustainable landscapes, providing greater social justice (cf. ISPN 2005). Agrarian reform can be carried out incorporating agroecology and agroforestry. Small farmers can make sustainable use of biodiversity, such as native fruits and nuts, handicrafts, honey and medicinal plants, as is done in the *Programa de Pequenos Projetos Ecossociais* (PPP-ECOS), supported by GEF-SGP (ISPN 2006). There are ample supplies of natural resources and labour as well as consumer markets in Brazil and abroad, but marketing requires changes in the regulatory framework (health, environment, registration and taxation), which blocks entry into formal markets. Another approach would be to include compensation for socioenvironmental services at the ecosystem level, not as privatized, pinpointed commodities, as is common in the Clean Development Mechanism of the Kyoto Protocol. This could be done through policies that favour small farmers, such as minimum prices for their products.

Market approaches should not be limited to certification, fair trade and carbon credits, which usually end up being very limited in scope and discriminating against poor and traditional communities. An alterative approach is to establish eco-social criteria for buyers, the most important of which would be to avoid new clearing anywhere. Developed countries could open their markets to sustainable products, buying good wood, non-timber forest products and non-forest sustainable products from communities.

Brazilian biomes are increasingly essential to the planet. International cooperation should go beyond establishing protected areas, which has been aimed at saving species, but has not always been effective (Hayes 2006), to adopt an ecosystem or landscape approach. Forest bias, ignoring or even sacrificing biomes with few trees, and fixation on the Amazon, as if other biomes were invisible or unimportant, should be overcome. In addition to financial and technical cooperation, there is need for advice on public policy, a change that depends on scientific inputs and public perceptions.

The responses to climate change and appropriate reactions to these responses involve crafting of appropriate policies, including scientific and technological policies, not limited to one biome, but reaching far beyond. Appropriate policies depend on investment in generating the knowledge needed, especially in universities engaged in ‘sustainability sciences’ (Clark & Dickson 2003), and on its appropriation by society. The key elements are research about causal relationships and caution in crafting policy. Generating and disseminating environmental and social knowledge for public policy and political action require both national responses and international cooperation from the countries that have been most responsible for the accumulation of greenhouse gases and are now responsible for vast ecological footprints, reaching deep into the Amazon and the Cerrado.

## Endnotes

1 Rather than ‘savannization’ that is only meant to refer to climate and is pejorative with regards to the neighboring Cerrado biome, the richest savannah in the world, it would be preferable to refer to ‘scrubification’, as suggested by Daniel Nepstad, or ‘*pecuarização’*, as suggested by this author, to describe the moribund forest and the expansion of cattle raising that actually happens on the ground.

2 The author has collected and filed more than 300 documents on the subject since October 2006, only a few of which, due to space limitations, are cited here.
